# Candidemia in a major regional tertiary referral hospital – epidemiology, practice patterns and outcomes

**DOI:** 10.1186/s13756-017-0184-1

**Published:** 2017-03-11

**Authors:** Jocelyn Qi-Min Teo, Samuel Rocky Candra, Shannon Jing-Yi Lee, Shannon Yu-Hng Chia, Hui Leck, Ai-Ling Tan, Hui-Peng Neo, Kenneth Wei-Liang Leow, Yiying Cai, Rachel Pui-Lai Ee, Tze-Peng Lim, Winnie Lee, Andrea Lay-Hoon Kwa

**Affiliations:** 1 Department of Pharmacy, Singapore General Hospital, Blk 8 Level 2, Outram Road, Singapore, 169608 Singapore; 2 Department of Microbiology, Singapore General Hospital, Outram Road, Singapore, 169608 Singapore; 3 Department of Pharmacy, National University of Singapore, 18 Science Drive 4, Singapore, 117543 Singapore; 4 SingHealth Duke-NUS Medicine Academic Clinical Programme, 20 College Rd, Singapore, 169856 Singapore; 5 Emerging Infectious Diseases, Duke-NUS Medical School, 8 College Rd, Singapore, 169857 Singapore; 6 Present address: Tan Tock Seng Hospital, 11 Jalan Tan Tock Seng, Singapore, 308433 Singapore

**Keywords:** *Candida*, Bloodstream infections, Antifungal susceptibility, *fks*, Mortality

## Abstract

**Background:**

Candidemia is a common cause of nosocomial bloodstream infections, resulting in high morbidity and mortality. This study was conducted to describe the epidemiology, species distribution, antifungal susceptibility patterns and outcomes of candidemia in a large regional tertiary referral hospital.

**Methods:**

A retrospective surveillance study of patients with candidemia was conducted at Singapore General Hospital between July 2012 and December 2015. In addition, incidence densities and species distribution of candidemia episodes were analysed from 2008 to 2015.

**Results:**

In the period of 2012 to 2015, 261 candidemia episodes were identified. The overall incidence was 0.14/1000 inpatient-days. *C. glabrata* (31.4%), *C. tropicalis* (29.9%), and *C. albicans* (23.8%) were most commonly isolated. The incidence of *C. glabrata* significantly increased from 2008 to 2015 (Coefficient 0.004, confidence interval 0–0.007, *p* = 0.04). Fluconazole resistance was detected primarily in *C. tropicalis* (16.7%) and *C. glabrata* (7.2%). *fks* mutations were identified in one *C. albicans* and one *C. tropicalis*. Candidemia episodes caused by *C. tropicalis* were more commonly encountered in patients with haematological malignancies (*p* = 0.01), neutropenia (*p* < 0.001) and higher SAPS II scores (*p* = 0.02), while prior exposure to echinocandins was associated with isolation of *C. parapsilosis* (*p* = 0.001). Echinocandins (73.3%) were most commonly prescribed as initial treatment. The median (range) time to initial treatment was 1 (0–9) days. The 30-day in-hospital mortality rate was 49.8%. High SAPS II score (Odds ratio, OR 1.08; 95% confidence interval, CI 1.05–1.11) and renal replacement therapy (OR 5.54; CI 2.80–10.97) were independent predictors of mortality, while drain placement (OR 0.44; CI 0.19–0.99) was protective.

**Conclusions:**

Decreasing azole susceptibilities to *C. tropicalis* and the emergence of echinocandin resistance suggest that susceptibility patterns may no longer be sufficiently predicted by speciation in our institution. Candidemia is associated with poor outcomes. Strategies optimising antifungal therapy, especially in the critically-ill population, should be explored.

## Background

*Candida* species are the leading cause of invasive fungal infections and a common cause of hospital-acquired bloodstream infections [1]. Candidemia has a profound impact on patient outcomes and the burden has increased significantly over the years. The crude mortality is high, ranging from 30–50% [2–4]; while the attributable mortality due to candidemia varied from 15–49% [5, 6]. Increasing reports of antifungal resistance, even in newer agents such as the echinocandins, further escalate the complexity in the management of candidemia [7].

Knowledge of antifungal susceptibility patterns is imperative in the selection of early and appropriate antifungal agents for improved patient outcomes. The variable epidemiology of candidemia, contributed by the geographical and temporal variations in incidence and species distribution [4, 8–10], underscores the continuing need for local surveillance of *Candida* species distribution and susceptibility patterns.

Furthermore, the introduction of new echinocandins into Singapore such as anidulafungin in 2008 and micafungin in 2013, coupled with the exponential increase in echinocandin usage in our institution for the past 5 years, suggest that current susceptibility patterns should be reviewed. A recent study has also reported the emergence of echinocandin resistance in the Asia-Pacific region [11]. The objectives of this study were 1) to investigate the incidence, species distribution and antifungal susceptibilities of candidemia, and 2) to describe the clinical features and outcomes of candidemia in our population.

## Methods

### Study setting and design

A retrospective surveillance study of patients with candidemia was conducted at Singapore General Hospital (SGH) between July 2012 and December 2015. SGH is the largest acute care hospital (1800 beds) in the country, and covers a wide range of medical and surgical specialties. The hospital is the national/regional referral centre for services such as plastic surgery and burns, renal medicine, nuclear medicine, pathology and haematology. SGH accounts for approximately 25% of the total acute hospital beds in the public sector and 20% of acute beds nationwide.

All adult inpatients (at least 21 years old) with ≥ 1 positive blood culture for *Candida spp.* were included into the study. Each positive *Candida* culture must be accompanied with temporally-related clinical signs and symptoms of infection for inclusion into the study. For each patient, only the first candidemia episode was recorded, unless the positive blood culture was obtained ≥ 30 days (with blood culture clearance and resolution of clinical features of infection of the first episode) or involved a different *Candida spp*. isolated from blood culture obtained ≥ 7 days after the first episode. Episodes involving > 1 *Candida spp.* isolated within 7 days of the first episode, defined as “mixed candidemia”, were regarded as a single episode.

### Microbiology and antifungal susceptibility testing

*Candida spp.* were isolated from blood using BD BACTEC™ FX (Becton, Dickinson and Company, Sparks, MD). The species were identified using MALDI Biotyper (BrukerDaltonik GmbH, Germany), morphology studies on cornmeal Tween 80 agar, and API 20C AUX (Biomerieux, Marcy l’Etoile, France). Isolates were stored in Microbank^TM^ storage vials (Pro-Lab Diagnostics, Round Rock, TX, USA) at −70 °C until testing.

Antifungal susceptibility testing was performed using Sensititre YeastOne® YO10 panel (Trek Diagnostics System, West Sussex, England) according to manufacturer’s recommendations. Minimum inhibitory concentrations (MICs) for amphotericin B, anidulafungin, caspofungin, micafungin, fluconazole, voriconazole, itraconazole, posaconazole and flucytosine were recorded. *Candida krusei* (*Issatchenkia orientalis*) ATCC 6258 and *C. parapsilosis* ATCC 22019 (American Type Culture Collection, Manassas, Virginia) were used as quality controls.

MICs were interpreted according to the current species-specific clinical breakpoints provided by the Clinical and Laboratory Standards Institute (CLSI) M27-S4 document [12]. Where clinical breakpoints were not available, the epidemiological cut-off values (ECV) were used to classify the isolates into wild-type or non-wild-type populations [13–15].

### Detection of *fks* mutations

Isolates classified as intermediate or resistant to echinocandins were tested for the presence of mutations in the *fks* genes. Hot spots 1 and 2 regions of *fks1* and *fks2* (for *C. glabrata* only) genes were amplified using polymerase chain reaction (PCR), as described previously [16].

### Clinical data collection

Clinical characteristics of patients with candidemia were obtained from inpatient charts and electronic medical records using a standardised case report form. Data extracted included demographics, hospitalisation history (previous hospital stay, previous intensive care unit (ICU) stay, length of hospital stay prior to candidemia), underlying medical conditions and prior exposure to invasive interventions (central lines, urinary catheters, drainage devices, invasive ventilation, dialysis, invasive surgery, total parenteral nutrition) and medical therapy (chemotherapy, immunosuppressive therapy, antibiotics, antifungal agents) within 30 days before the first positive blood culture. Charlson comorbidity index at the time of admission and Simplified Acute Physiology Score (SAPS) on the day of the first positive blood culture were also recorded. Information on the management of candidemia (choice and duration of antifungal agents) and outcome (in-hospital all-cause mortality within 30 days) were collected.

### Data and statistical analyses

To calculate and analyse the incidence of candidemia, the number of candidemia episodes were obtained from the clinical microbiology laboratory computerised database, while inpatient-days were obtained from the hospital administrative database. Incidence data was available from 2008, hence trend analyses were performed for the period from 2008 to 2015. Incidence rates were calculated as the number of candidemia episodes per 1000 inpatient-days. Linear regression was used to determine trends over time in the incidences of candidemia.

Categorical variables were presented as numbers and percentages; and were compared using the Χ^2^ or Fisher’s exact test, as appropriate. Continuous variables were presented as mean ± SD or median and range; and were compared using the Student’s *t* test, Mann–Whitney test, or Kruskal-wallis test, depending on the validity of the normality assumption.

A multivariable logistic regression model was used to identify predictors associated with 30-day mortality. Clinically plausible variables identified in the bivariate analysis were included in the multivariable logistic regression model if *p* < 0.1. Significant factors which may covary were grouped and only one factor from each group was selected for entry into the model. The final model was chosen on the basis of biologic plausibility. Odds ratios (OR) and 95% confidence intervals (CI) were calculated to evaluate the strength of any association. For all calculations, a 2-tailed *p* value of less than 0.05 was considered to reveal a statistical significant difference. Statistical analyses were performed using IBM SPSS Statistics for Windows, Version 23.0 (IBM Corp., Armonk, NY).

## Results

### Incidence and species distribution

From 2012 to 2015, 261 candidemia episodes involving 254 patients and 272 isolates were analysed. Seven patients had two separate episodes each with distinct *Candida* species, while a patient had a repeated episode involving the same *Candida* species. The incidence was 0.14 episodes per 1000 inpatient-days during the study period. *C. glabrata* (82/261, 31.4%), *C. tropicalis* (78/261, 29.9%), *C. albicans* (62/261, 23.8%), and *C. parapsilosis* (36/261, 13.8%) accounted for majority of the episodes. Other species including *C. dubliniensis* (*n* = 7), *C. krusei* (*n* = 3), *C. guilliermondii (Meyerozyma guilliermondii)* (*n* = 1), *C. kefyr (Kluyveromyces marxianus)* (*n* = 1), *C. haemulonis* (*n* = 1) and *C. pseudohaemulonii* (*n* = 1) accounted for the remaining episodes. Of these 261 episodes, 11 (4.2%) were mixed candidemia episodes*.*

**Fig 1:**
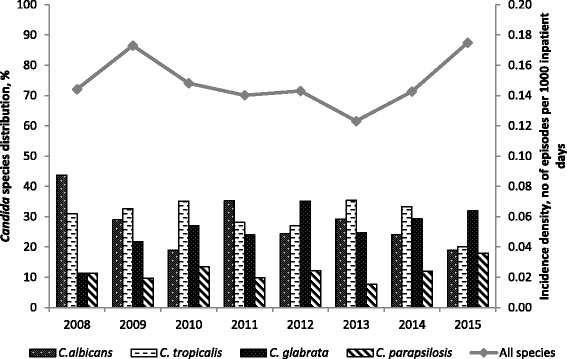
Incidence densities of candidemia episodes and distribution of *Candida* species from 2008 to 2015

The incidence density and species distribution are displayed in Fig. 1. The overall incidence density was 0.15 (range 0.12–0.18) episodes/1000 inpatient-days and 0.89 (range 0.74–1.05) episodes/1000 admissions from 2008 to 2015. Analysing the incidence densities from 2008 to 2015, we found no significant change in the incidence density of candidemia [Coefficient 0.00009, confidence interval (CI) - 0.007–0.007, *p* = 0.98]. However, we did note that the overall incidence density increased from 0.14 in 2014 to 0.18 episodes/1000 inpatient-days in 2015, suggesting the need for continual monitoring. There was a significant increasing trend in the incidence density of *C. glabrata* (Coefficient 0.004, CI 0–0.007, *p* = 0.04)*,* while the incidence densities of the other *Candida spp.* remained stable. The proportions of *C. glabrata* increased from 11.3% in 2008 to 31.6% in 2015 and that of *C. albicans* decreased from 44% in 2008 to 19% in 2015.

### Antifungal susceptibilities

Antifungal susceptibilities were available for 271 isolates, except for one *C. parapsilosis* (Table 1). Among isolates with available clinical breakpoints, overall susceptibility rates were 59.5% (153/257) for fluconazole, 86.9% (152/175) for voriconazole, 99.2% (255/257) for anidulafungin, 98.1% (252/257) for caspofungin and 98.9% (254/257) for micafungin. Using the clinical breakpoints, *C. albicans* and *C. parapsilosis* retained high susceptibility (>94%) to fluconazole and voriconazole. However, more than 20% of the *C. tropicalis* isolates were non-susceptible to fluconazole and voriconazole. The proportions of isolates classified as wild-type (MIC value less than or equals to ECV) for fluconazole, voriconazole, itraconazole and posaconazole were similar among *C. albicans*, *C. glabrata* and *C. parapsilosis* (ranged from 94–100%). Decreased susceptibilities (non wild-type; MIC value greater than ECV) to fluconazole and voriconazole were prominent in *C. tropicalis* isolates. Echinocandin resistance was rare, occurring only in three isolates (*C. albicans* = 1; *C. tropicalis* =1 and *C. glabrata* = 1) when assessed using both clinical breakpoints and ECVs. Most isolates had amphotericin B and flucytosine MICs below ECVs (96–100%), although a number of *C. parapsilosis* were classified as non-wild-type (20%). The amphotericin B MICs of these non-wild-type isolates were 2 μg/mL, which were just one dilution above the ECV (1 μg/mL) utilised in this study. Furthermore, the ECV used in this study was derived using the YeastOne® method and is one dilution lower than the ECVs for the other species (2 μg/mL) and the ECV derived from broth dilution methods.

**Table 1:** Antifungal susceptibilities of major species of *Candida* isolates^a^

Antifungal	MIC_50_ (μg/mL)	MIC_90_ (μg/mL)	MIC Range (μg/mL)	%S^b^	%SDD/I^b^	%R^b^	%WT^c^
*C albicans (n = 62)*							
Fluconazole	0.5	2	≤0.12–>256	95.2	1.6	3.2	93.5
Itraconazole	0.06	0.12	≤0.015–>16	–	–	–	96.7
Posaconazole	0.015	0.06	≤0.08–>8	–	–	–	96.7
Voriconazole	≤0.008	0.03	≤0.008–>8	93.6	3.2	3.2	93.5
Anidulafungin	≤0.015	0.03	≤0.015–0.25	100	0	0	98.4
Caspofungin	0.03	0.06	0.015–4	98.4	0	1.6	98.4
Micafungin	≤0.008	0.015	≤0.008–2	98.4	0	1.6	98.4
Flucytosine	≤0.06	0.25	≤0.06–>64	–	–	–	96.7
Amphotericin B	0.5	1	≤0.12–1	–	–	–	100
*C. glabrata* (*n* = 82)							
Fluconazole	16	32	1–>256	–	92.8	7.2	97.6
Itraconazole	1	1	0.12–>16	–	–	–	93.9
Posaconazole	2	2	0.12–>8	–	–	–	95.1
Voriconazole	0.5	2	0.03–>8	–	–	–	97.6
Anidulafungin	0.03	0.06	≤0.015–4	98.8	0	1.2	98.7
Caspofungin	0.12	0.12	0.03–>8	96.4	2.4	1.2	96.3
Micafungin	0.015	0.015	≤0.008–4	98.8	0	1.2	98.7
Flucytosine	≤0.06	0.12	≤0.06–0.25	–	–	–	100
Amphotericin B	1	1	0.25–2	–	–	–	100
*C. tropicalis* (*n* = 78)							
Fluconazole	2	64	0.5–>256	78.2	5.1	16.7	84.6
Itraconazole	0.25	0.5	0.03–>16	–	–	–	96.1
Posaconazole	0.12	0.5	0.03–4	–	–	–	98.7
Voriconazole	0.12	4	≤0.008–>8	75.6	11.5	12.8	80.8
Anidulafungin	0.03	0.12	≤0.015–0.5	98.7	1.3	0	98.7
Caspofungin	0.03	0.06	0.015–2	98.7	0	1.3	98.7
Micafungin	0.03	0.03	≤0.008–1	98.7	0	1.3	98.7
Flucytosine	≤0.06	0.12	≤0.06–32	–	–	–	96.2
Amphotericin B	1	1	0.25–2	–	–	–	100
*C. parapsilosis* (*n* = 35)							
Fluconazole	0.5	2	0.25–4	97.1	2.9	0	100
Itraconazole	0.06	0.06	≤0.015–0.12	–	–	–	100
Posaconazole	0.03	0.06	0.015–0.12	–	–	–	100
Voriconazole	0.015	0.03	≤0.008–0.6	100	0	0	97.1
Anidulafungin	0.5	2	0.12–2	100	0	0	100
Caspofungin	0.25	0.5	0.06–1	100	0	0	100
Micafungin	0.5	2	0.12–2	100	0	0	100
Flucytosine	≤0.06	0.5	≤0.06–1	–	–	–	100
Amphotericin B	1	2	0.25–2	–	–	–	80.0

*S* susceptible, *SDD* susceptible dose-dependent, *I* intermediate, *R* resistant, *WT* wild-type

^a^MICs are only reflected for the predominant species

^b^Susceptibilities were assessed based on CLSI species-specific clinical interpretative breakpoints [12]. Clinical breakpoints are not available for itraconazole, posaconazole, flucytosine and amphotericin B for all species and voriconazole for *C. glabrata*

^c^ECVs were derived from [13, 14] and [15]

*fks* mutations were detected in the echinocandin-resistant *C. albicans* (caspofungin MIC 4 μg/mL; anidulafungin MIC 0.25 μg/mL; micafungin MIC 2 μg/mL) and *C. tropicalis* (caspofungin MIC 2 μg/mL; anidulafungin MIC 0.5 μg/mL; micafungin 1 μg/mL) isolates. Both isolates harboured a point mutation (S645P in *C. albicans* and S80P in *C. tropicalis*) in the hotspot 1 region of the *fks1* gene. The two isolates remained susceptible to all other antifungals. Interestingly, *fks* mutations were not identified in the *C. glabrata* isolate which was resistant (caspofungin MIC ≥ 8 μg/mL; anidulafungin MIC 4 μg/mL; micafungin MIC 4 μg/mL).

### Clinical characteristics

The clinical characteristics of the candidemia episodes are summarised in Table 2. The median age of patients with candidemia was 65 years and incidence did not differ by gender (52.9% male *vs*. 47.1% female, *p* = 0.59). The episodes occurred primarily in the medical wards (42.1%), followed by intensive care units (ICUs) (38.3%), surgical wards (19.5%). Patients admitted to haematology-oncology (19.9%), internal medicine (19.5%) and general surgery units (12.3%) encountered the most episodes.

**Table 2:** Clinical characteristics of candidemia episodes

	All	*C. glabrata*	*C. tropicalis*	*C. albicans*	*C. parapsilosis*	*p*
	*n* = 261	*n = 75 (28.6%)*	*n* = 71 (27.1%)	*n = 59 (22.6%)*	*n = 33 (12.6%)*	
Demographics						
Male sex	138 (52.9)	37 (49.3)	39 (54.9)	32 (54.2)	22 (66.7)	0.42
Median age (range)	65 (22–101)	67 (24–95)	63 (28–90)	68 (27–101)	61 (28–86)	0.06
Ward type						0.83
Medical ward	110 (42.1)	30 (40.0)	35 (49.3)	23 (39.0)	14 (42.4)	
Surgical ward	51 (19.5)	16 (21.3)	10 (14.1)	14 (23.7)	7 (21.2)	
ICU	100 (38.3)	29 (38.7)	26 (36.6)	22 (37.3)	12 (36.4)	
Elective admission	27 (10.3)	12 (16.0)	5 (7.0)	5 (8.5)	4 (12.1)	0.32
Comorbidities						
Malignancies	106 (40.6)	34 (45.3)	29 (40.8)	23 (39.0)	12 (36.4)	0.81
Haematological	27 (10.3)	3 (4.0)	13 (18.3)	6 (10.2)	2 (6.1)	***0.03***
Oncological	84 (32.2)	32 (42.7)	17 (23.9)	18 (30.5)	11 (33.3)	0.11
With metastases	36 (13.8)	16 (21.3)	11 (15.5)	6 (10.2)	3 (9.1)	0.23
Diabetes	103 (39.5)	31 (41.3)	25 (35.2)	23 (39.0)	12 (36.4)	0.89
Chronic renal failure	67 (25.7)	17 (22.7)	22 (31.0)	14 (23.7)	8 (24.2)	0.67
Hepatobiliary disorders	58 (22.2)	17 (22.7)	20 (28.2)	8 (13.6)	8 (24.2)	0.25
Myocardial infarction	43 (16.5)	10 (13.3)	13 (18.3)	15 (25.4)	1 (3.0)	***0.04***
Cerebrovascular disease	29 (11.1)	12 (16.0)	8 (11.3)	4 (6.8)	4 (12.1)	0.44
Median (range) Charlson score	5 (0–15)	6 (0–15)	5 (0–14)	4 (0–12)	4 (0–9)	0.08
Risk factors						
Central venous catheter	192 (73.6)	47 (62.7)	55 (77.5)	46 (78.0)	26 (78.8)	0.11
Drain	60 (23.0)	22 (29.3)	14 (19.7)	16 (27.1)	6 (18.2)	0.43
Mechanical ventilation	111 (42.5)	26 (34.7)	31 (43.7)	25 (42.4)	16 (48.5)	0.52
Total parenteral nutrition	52 (19.9)	12 (16.0)	13 (18.3)	12 (20.3)	10 (30.3)	0.37
Surgery	170 (65.1)	51 (68.0)	44 (66.0)	39 (66.1)	20 (60.6)	0.83
Gastrointestinal surgery	41 (15.7)	18 (24.0)	5 (7.0)	9 (24.3)	5 (15.2)	0.05
Renal replacement therapy	85 (32.6)	16 (21.3)	28 (39.4)	21 (35.6)	12 (36.4)	0.10
Antimicrobial therapy	236 (90.4)	67 (89.3)	68 (95.8)	53 (89.8)	27 (81.8)	0.15
Antifungal therapy	51 (19.5)	13 (17.3)	15 (21.1)	8 (13.6)	11 (33.3)	0.13
Azole	24 (9.2)	5 (6.7)	10 (14.1)	6 (10.2)	2 (6.1)	0.41
Echinocandin	30 (11.5)	8 (10.7)	6 (8.5)	2 (3.4)	10 (30.3)	***0.001***
Immunosuppressive therapy	76 (29.1)	17 (22.7)	28 (39.4)	16 (27.1)	10 (30.3)	0.16
Neutropenia	21 (8.0)	3 (4.0)	13 (18.3)	2 (3.4)	2 (6.1)	***0.004***
Therapy						
Primary therapy						0.15
Echinocandin	165 (73.3)	45 (76.3)	49 (81.7)	32 (60.4)	22 (71.0)	
Azole	52 (23.1)	12 (20.3)	11 (18.3)	17 (32.0)	8 (25.8)	
Others	8 (3.1)	2 (3.3)	1 (1.7)	4 (6.8)	1 (3.2)	
None	36 (13.8)	16 (21.3)	10 (14.1)	6 (10.2)	2 (6.1)	
Median (range) time to primary therapy, days	1 (0–9)	2 (0–7)	1 (0–3)	2 (0–5)	1 (0–9)	***0.01***
Median (range) duration of therapy, days	15 (1–140)	16 (2–61)	11 (1–96)	16 (1–140)	15 (2–47)	***0.01***
Infection Characteristics & Outcomes						
Median (range) time to positive culture, days	12 (0–282)	11 (0–282)	14 (0–123)	14 (0–79)	37 (0–104)	0.37
Median (range) time to reporting positive culture, days	2 (0–10)	3 (0–9)	1 (0–10)	2 (1–5)	2(1–3)	***<0.001***
Median (range) time to species identification, days	5 (2–22)	6 (2–12)	4 (2–12)	5 (2–9)	5 (3–7)	***<0.001***
Median (range) SAPS II score	49 (14–103)	48 (18–95)	55 (18–93)	48 (23–103)	44 (14–72)	***0.01***
Median (range) Pitts' bacteraemia score	3 (0–14)	2 (0–11)	3 (0–12)	3 (0–11)	2 (0–8)	0.86
Severe sepsis at time of culture	151 (57.9)	49 (65.3)	43 (60.6)	33 (55.9)	13 (58.0)	0.08
ICU stay	131 (50.2)	36 (48.0)	35 (49.3)	30 (50.8)	16 (48.5)	0.99
Concurrent infection	127 (48.7)	33 (44.0)	36 (57.0)	30 (50.8)	16 (48.5)	0.83
Candida colonization/infection at other sites	118 (45.2)	41 (54.7)	39 (54.9)	34 (57.6)	18 (54.5)	0.99
30-day in-hospital all-cause mortality	130 (49.8)	38 (50.7)	42 (59.2)	28 (47.5)	9 (27.3)	***0.03***

All variables are denoted as number of patients with the characteristic or belonging to the category [n (%)], unless otherwise stated

Sub-group analyses are shown only for episodes involving major *Candida spp.* and not for mixed candidemia and less common species

Comorbidities < 10% in occurrence are not reflected

Significant variables are reflected in bold and italics

Most of the patients presented with multiple comorbidities (median Charlson score = 5, range 0–15), with many having malignancies (40.6%). Diabetes was also common among these patients (39.5%). Prior antibiotic exposure (90.4%), central venous catheter placement (73.6%), and surgery (65.1%) were common risk factors. A large number of patients were colonised or infected with *Candida* at other non-blood sites (45.2%) and had concurrent bacterial infections (48.7%). In addition, it appears that candidemia episodes caused by *C. tropicalis* were more commonly encountered in patients with haematological malignancies (*p* = 0.01), neutropenia (*p* < 0.001) and higher SAPS II scores (*p* = 0.02). Exposure to echinocandins was also associated with candidemia episodes caused by *C. parapsilosis* (*p* = 0.001).

### Antifungal therapy and outcomes

Antifungal therapy was initiated in 225 (86.2%) episodes (Table 2). All but six of the 36 patients who did not receive treatment died before blood cultures flagged positive. Treatment was not initiated in four patients as they were conservatively managed. Interestingly, physicians elected not to initiate treatment in the remaining two patients.

Echinocandins were the initial treatment of choice (73.3%), followed by azoles (23.1%). Caspofungin (93.4%) was more commonly used, since it was the only echinocandin in the formulary until anidulafungin’s inclusion in August 2015. Among the patients receiving treatment, 32 (14.2%) were already receiving antifungals as prophylaxis or empiric treatment on the day which cultures were taken. Fluconazole was the only azole used as initial treatment of candidemia in our institution. The median (range) time to initial treatment was 1 (0–9) days. Treatment was initiated in 73 (32.4%) patients on day of culture and in 172 (76.4%) patients within two days. The median (range) duration of therapy was 15 (1–140) days.

Patients with candidemia were moderately to severely-ill – 57.9% were having severe sepsis and the median (range) SAPS II score was 49 (14–103) at the time of culture. Many of these episodes (38.3%) occurred in critically-ill patients warded in the ICUs. We also observed that some patients (11.9%), who were initially in the general wards at the time of culture, required admission into the ICU after *Candida* isolation, suggesting that candidemia episodes can result in severe illness. Mortality occurred in 150 (57.4%) episodes during the admission. The 7-day, 14-day and 30-day in-hospital mortality rates were 28.3%, 39.8%, and 49.8%. The mortality rate was lowest in patients infected with *C. parapsilosis* (23.5%) (*p* = 0.03). Among the 225 patients who received treatment, the 30-day in-hospital mortality rate was 41.4%, while all but two (94.4%) of the non-treated episodes resulted in death.

### Predictors of mortality

The characteristics of survivors and non-survivors at 30 days are depicted in Table 3. Based on the multivariable logistic regression model, high SAPS II score (Odds ratio, OR 1.08; 95% confidence interval, CI 1.06–1.11) and renal replacement therapy (OR 4.31; CI 2.24–8.28) were the only factors associated with 30-day mortality. Presence of drains was a protective factor (OR 0.45; CI 0.21–0.94). Mortality occurred rapidly in many of the non-survivors, hence receipt/type of antifungal therapy was not included in this model, since antifungal therapy could not be initiated in this subset of patients. To examine the impact of initial antifungal therapy on 30-day mortality, a separate analysis was performed for candidemia episodes where treatment was administered. Results were similar when non-treated episodes were excluded. High SAPS II score, renal replacement therapy and drains placement were significant factors in the multivariable regression model (Table 4). The choice and timing of initial antifungal therapy was not associated with mortality.

**Table 3:** Characteristics of survivors vs. non-survivors

	Survivors	Non-survivors	*p*
	*n* = 134	*n* = 127	
Demographics			
Male sex	73 (54.5)	65 (51.2)	0.59
Median age (range)	64 (22–95)	65 (24–101)	0.81
Ward type			***<0.001*** ^***a***^
Medical ward	66 (49.3)	44 (34.6)	
Surgical ward	37 (27.6)	14 (11.0)	
ICU	31 (23.1)	69 (54.3)	
Elective admission	14 (10.4)	13 (10.2)	0.96
Comorbidities			
Malignancies	58 (43.3)	48 (51.6)	0.37
Diabetes	53 (39.6)	50 (39.4)	0.97
Chronic renal failure	22 (16.4)	45 (35.4)	***<0.001***
Hepatobiliary disorders	25 (18.7)	33 (26.0)	0.16
Myocardial infarction	19 (14.2)	24 (18.9)	0.30
Cerebrovascular disease	11 (8.2)	18 (14.2)	0.13
Median (range) Charlson score	4 (0–15)	5 (0–14)	0.09^a^
Median (range) SAPS II score	43 (14–82)	58 (27–103)	***<0.001*** ^a^
Risk factors			
Central venous catheter	89 (66.4)	103 (81.1)	***0.007*** ^a^
Drain	37 (27.6)	23 (18.1)	0.07^a^
Mechanical ventilation	47 (35.1)	64 (50.4)	***0.01*** ^a^
Total parenteral nutrition	28 (20.9)	24 (18.9)	0.69
Surgery	81 (60.4)	89 (70.1)	0.10
Gastrointestinal surgery	20 (14.9)	21 (16.5)	0.72
Renal replacement therapy	23 (17.2)	62 (48.8)	***<0.001*** ^a^
Antimicrobial therapy	116 (86.6)	120 (94.5)	0.30
Antifungal therapy	27 (20.1)	24 (18.9)	0.79
Immunosuppressive therapy	33 (24.6)	43 (33.9)	0.10
Neutropenia	10 (7.5)	11 (8.7)	0.72
Therapy			
Initial therapy			***<0.001*** ^***b***^
Echinocandin	89 (66.4)	76 (59.8)	
Azole	40 (29.9)	12 (9.4)	
Others (Amphotericin or combination)	3 (2.2)	5 (3.9)	
None	2 (1.5)	34 (26.8)	
Received initial therapy within 24 h	58 (43.2)	64 (50.4)	***<0.001*** ^***b***^
Infection Characteristics			
Species			***0.04*** ^a^
*C. albicans*	32 (23.9)	27 (21.3)	
*C. glabrata*	39 (29.1)	36 (28.3)	
*C. tropicalis*	29 (21.6)	42 (33.1	
*C. parapsilosis*	24 (17.9)	9 (7.1)	
Median (range) time to reporting positive culture, days	2 (0–10)	2 (0–10)	0.08^a^
Median (range) time to species identification, days	5 (2–16)	5 (2–22)	***0.001*** ^a^
Median (range) Candida score	2 (0–5)	3 (0–5)	***0.01***
Median (range) Pitts' bacteraemia score	2 (0–11)	5 (0–14)	***<0.001*** ^a^
Severe sepsis at time of culture	64 (47.8)	87 (68.5)	***0.001*** ^a^
Concurrent bacterial infection	59 (46.5)	68 (53.5)	0.12
Candida colonization/infection at other sites	61 (45.5)	57 (44.9)	0.92

All variables are denoted as number of patients with the characteristic or belong to the category n (%), unless otherwise stated

Significant variables are reflected in bold and italics

^a^Factors entered into multivariable logistic regression model

^***b***^Additional factors entered into multivariable logistic regression model including only treated episodes

**Table 4:** Multivariable logistic regression model for mortality in treated cases (*n* = 225)

Variable	OR (95% CI)
SAPS II score	1.08 (1.05–1.11)
Presence of drains	0.44 (0.19–0.99)
Renal replacement therapy	5.54 (2.80–10.97)

## Discussion

We report here a comprehensive epidemiological study of candidemia conducted at a large tertiary regional referral centre, which included the clinical characteristics, antifungal treatment, species distribution, antifungal susceptibilities and outcomes of candidemia. Our study showed that the incidence density of candidemia in our institution has remained fairly stable since 2008. This concurs with the general trend of stability in incidence reported in other developed countries, such as the United States and Europe [2, 17]. A recent study comparing candidemias among sites in Asia indicated that rates in Singapore (0.15 episodes per 1000 patient-days) were comparable with most other Asian countries, with the exception of Taiwan (0.37 per 1000 patient-days) and India (1.24 per 1000 patient-days) [10]. On a more global scale, our rates were lower than those in Italy (0.33 per 1000 patient-days) [18], and Brazil (0.37 per 1000 patient-days) [19]. It appears that the species distribution in our institution is changing. Previous local studies reported a predominance of *C. tropicalis*, a finding commonly observed in tropical regions [10, 20]. We observed an increasing proportion of *C. glabrata* from 11% in 2008 to 31% in 2015, overtaking *C. tropicalis* as the predominant species.

With respect to antifungal susceptibilities, while *C. albicans* and *C. parapsilosis* remained mostly susceptible, fluconazole resistant rates of *C. tropicalis* was 17%. Notably, the fluconazole MIC_90_ of *C. tropicalis* increased from 2 μg/mL in 2007 to 64 μg/mL reported in our study [20]. This MIC uptrend suggests that *C. tropicalis*, one of the predominant species in our context, is increasingly becoming less susceptible. Further molecular investigations are underway to understand the mechanisms related to azole resistance in these isolates.

Another noteworthy finding of our study was the emergence of echinocandin resistance in the Southeast Asia region. In the post-echinocandin era, there have been increasing reports of echinocandin treatment failures in most clinically-relevant species, especially in *C. glabrata* [7, 21–24]. Fortunately, resistance rates remained rare in the local context. There were only three (1.1%) isolates which were echinocandin-resistant, of which two had *fks* mutations. To the best of our knowledge, this is the first incidence of *fks* mutations in *Candida* bloodstream isolates other than *C. glabrata* identified locally. While the *fks* mutations identified in our isolates have been previously described, it is interesting to note that resistance developed rapidly (within 4 days of exposure to caspofungin) in one of the patients. Development in resistance has been primarily related to prolonged use of echinocandins, which was observed in the other patient, who had received 30 days of caspofungin prior to *Candida* isolation [22].

Our study observed a high 30-day mortality rate of 49%. Like many previous studies, we found that mortality was associated with severity of illness at onset of candidemia, suggesting that the poor outcomes of patients with candidemia is likely related to the poor prognosis of these patients with multiple comorbidities [25]. Receipt of renal replacement therapy was also associated with 30-day mortality. This could be an indication of the underlying organ dysfunction contributing to severity of illness. Drains placement prior to *Candida* isolation was found to be protective, suggesting that perhaps source control could contribute to better survival in patients with secondary candidemia.

Initial antifungal choice did not appear to be associated with mortality in our study. Although the Infectious Diseases Society of America guidelines have recommended the use of an echinocandin as a first-line agent, randomised controlled trials conducted so far have yet to conclusively demonstrate superiority of one agent over another [26–28]. A recent study has also illustrated that clinical severity, rather than initial antifungal strategy, was significantly correlated with mortality [25]. One reason why we were unable to detect any association of initial antifungal choice with mortality could be because we did not account for the appropriateness of the therapy in terms of dosing. Furthermore, pharmacokinetic variability can result in fluctuating antifungal levels in individual patients [29]. Perhaps, the impact of initial antifungal choice on treatment outcomes can be better elucidated if antifungal dosing was individualised, such as through the use of therapeutic drug monitoring. This therapeutic approach is currently being explored in our institution.

Although a large number of our patients received antifungals in a timely fashion, there was still a delay in therapy for some patients, with some receiving antifungals more than a week after cultures were taken. The time to administration of antifungals could be limited by the lack of rapid diagnostic tests available in our institution. It takes an average of two days to report a positive *Candida* blood culture, and in some instances even up to a week.

Our study was not without limitations. This was a single-centre study and our results might not be extrapolated to other institutions as the epidemiology of candidemia can be highly institution-specific. The retrospective nature of the study also precluded the analysis of impact of time of catheter removal on mortality. Nevertheless, this study provides important epidemiological findings which are instrumental in designing strategies for better management of candidemia in our institution.

## Conclusions

While incidence of candidemia appeared to be stable, incidence of *C. glabrata* is increasing. *C. glabrata* and *C. tropicalis* contributed to majority of the candidemia cases in our institution. Decreasing azole susceptibilities to *C. tropicalis* and the emergence of echinocandin resistance suggests that susceptibility patterns may no longer be sufficiently predicted by speciation in our institution. Routine antifungal susceptibility, particularly for *C. tropicalis*, might be essential to guide clinician to effectively manage patients with invasive *Candida* infections. Candidemia was associated with high mortality, and antifungal stewardship efforts in individualising antifungal dosing through therapeutic drug monitoring should be further explored to improve outcomes in this population.

## Abbreviations

ATCC: American Type Culture Collection
CI: Confidence interval
CLSI: Clinical and Laboratory Standards Institute
ECV: Epidemiological cut-off values
ICU: Intensive care unit
MIC: Minimum inhibitory concentration
OR: Odds ratio
PCR: Polymerase chain reaction
SAPS: Simplified acute physiology score
SGH: Singapore General Hospital
