# Correction to: Candidemia in a major regional tertiary referral hospital – epidemiology, practice patterns and outcomes

**DOI:** 10.1186/s13756-018-0413-2

**Published:** 2018-11-07

**Authors:** Jocelyn Qi-Min Teo, Samuel Rocky Candra, Shannon Jing-Yi Lee, Shannon Yu-Hng Chia, Hui Leck, Ai-Ling Tan, Hui-Peng Neo, Kenneth Wei-Liang Leow, Yiying Cai, Pui Lai Rachel Ee, Tze-Peng Lim, Winnie Lee, Andrea Lay-Hoon Kwa

**Affiliations:** 1 Department of Pharmacy, Singapore General Hospital, Blk 8 Level 2, Outram Road, Singapore, 169608 Singapore; 2 Department of Microbiology, Singapore General Hospital, Outram Road, Singapore, 169608 Singapore; 3 Department of Pharmacy, National University of Singapore, 18 Science Drive 4, Singapore, 117543 Singapore; 4 SingHealth Duke-NUS Medicine Academic Clinical Programme, 20 College Rd, Singapore, 169856 Singapore; 5 Emerging Infectious Diseases, Duke-NUS Medical School, 8 College Rd, Singapore, 169857 Singapore; 6 Present Address: Tan Tock Seng Hospital, 11 Jalan Tan Tock Seng, Singapore, 308433 Singapore

## Correction

The original article [1] contains an error whereby the author, Pui Lai Rachel Ee’s name is incorrectly presented. The correct presentation of the name can be viewed in this Correction article.
